# Supplementary material for: Efficient plant genome engineering using a probiotic sourced CRISPR-Cas9 system
Source: Nat Commun. 2023 Sep 29;14:6102. doi: 10.1038/s41467-023-41802-9 (PMC10541446; doi:10.1038/s41467-023-41802-9)
Supplement: Supplementary file 1 — Supplementary Information [file 41467_2023_41802_MOESM1_ESM.pdf]

Efficient plant genome engineering using a probiotic sourced CRISPR-Cas9 system

Zhong *et al.*

Supplementary Table 1. Summary of LrCas9 mediated singular genome editing in rice T_0 lines.

Target gene	Protospacer+PAM	Tested T0 lines	Mutated T0 lines (number; ratio)	Biallelic T0 lines (number; ratio)
OsPDS	AGTCCTGGCAAACAACCTGCAGAAA	20	11; 55.0%	2; 10.0%
OsPDS	TGGCATTTCTACCTTATCGATGAAA	18	9; 50.0%	2; 11.0%
OsDEP1	TCCCGAGCGCGGAGTACGTACGAAA	20	17; 85.0%	5; 40.0%
OsBADH2	GCACCTGTCTCTCTTCCAATGGAAA	18	8; 44.4%	2; 10.5%
Os03g0568400	TCGACCTCTGGAAGATGGAAGGAAA	18	7; 38.9%	2; 10.5%
Os03g0603100	GAGCGCCTCGGCATGTCCCTCGAAA	18	3; 16.7%	2; 11.1%

Supplementary Table 2. Additional predicted off-target sites by CRISPR-GE.

	Index	Chromosome	Position	Method	Sequence	PAM	Gene_ID	Region	Edited
Os-AG04	Off-target9	Chr04	7973580	CRISPR-GE	AGCTCTGGAAAACACCCTGC	AGAAA	LOC_Os04g14220	3'-UTR	No
	Off-target10	Chr11	6884289	CRISPR-GE	AGCTGTGGGAAGCAACCTGC	AGAAA	LOC_Os11g12330	CDS	No
	Off-target11	Chr07	26745484	CRISPR-GE	AGTCATGGCTAACCAGCTGC	AGATA	-	intergenic	No
	Off-target12	Chr08	17584250	CRISPR-GE	AATCCTGGCCAACAACCTTT	AAAAA	-	intergenic	No
Os-TG02	Off-target9	Chr06	4789241	CRISPR-GE	TGGCCTTTCTTCCTTATAGA	ACAAT	-	intergenic	No
	Off-target10	Chr02	16472623	CRISPR-GE	TTGCATATTTACCGTATCGA	AAAAA	LOC_Os02g27820	intron	No
	Off-target11	Chr02	17106320	CRISPR-GE	TAGTATCTCTCCCTTATCCA	AGAAA	-	intergenic	No
	Off-target12	Chr01	11274385	CRISPR-GE	TAGCAATTATACCTTATTGC	AGAAA	LOC_Os01g19870	intron	No
	Off-target13	Chr08	23272375	CRISPR-GE	TGGCATTGCTACAGTGTTGA	AGAAA	-	intergenic	No

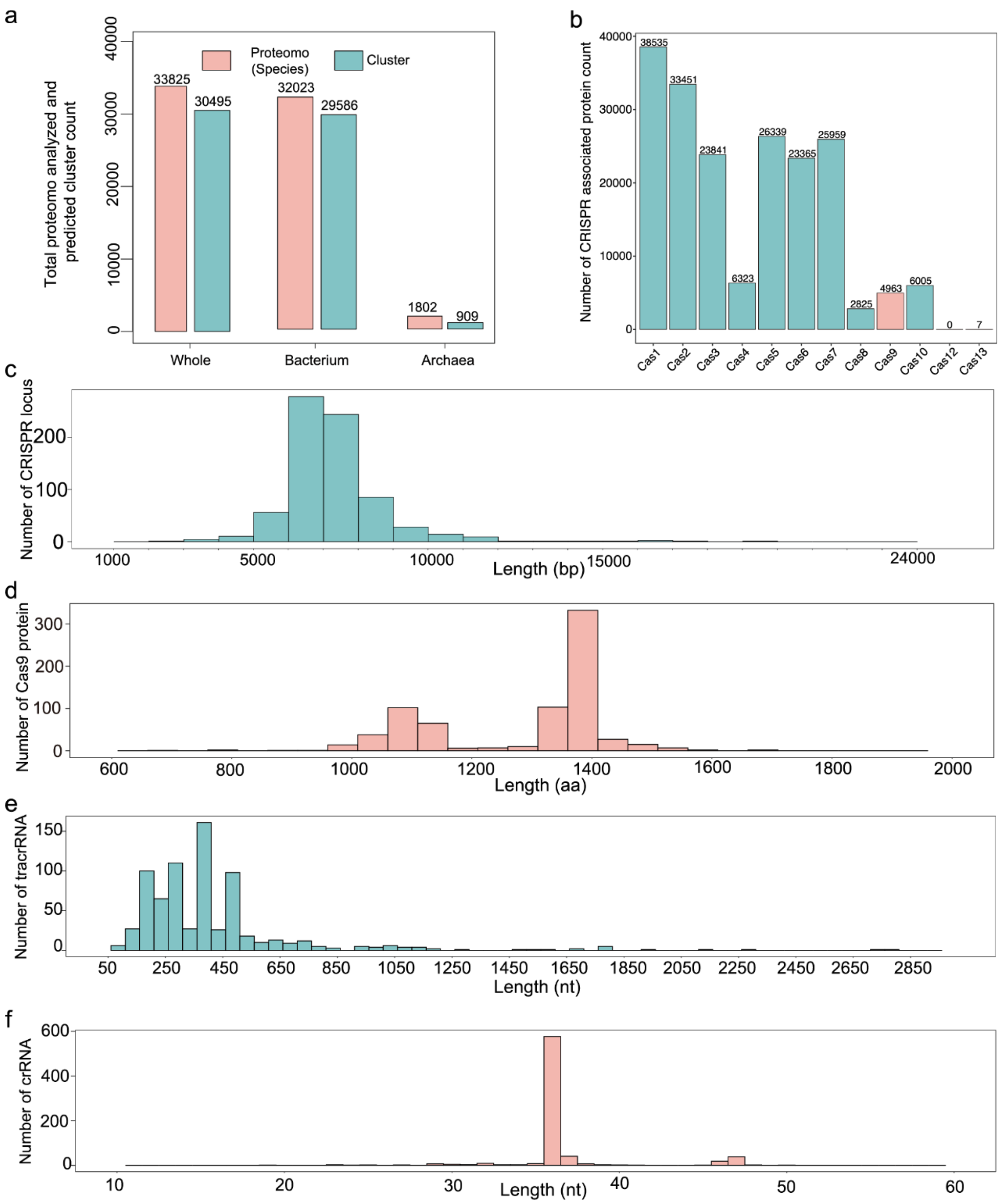

Supplementary Fig. 1. *In-silico* analysis of putative CRISPR-Cas9 systems. a, Total proteomes and clusters analyzed in this study. **b,** Total CRISPR associated proteins identified in this study. **c,** The DNA lengths of identified CRISPR-Cas9 systems in this study. **d,** The protein lengths of identified Cas9 proteins in this study. **e,** The identified tracrRNA lengths in this study. **f,** The identified crRNA lengths in this study.

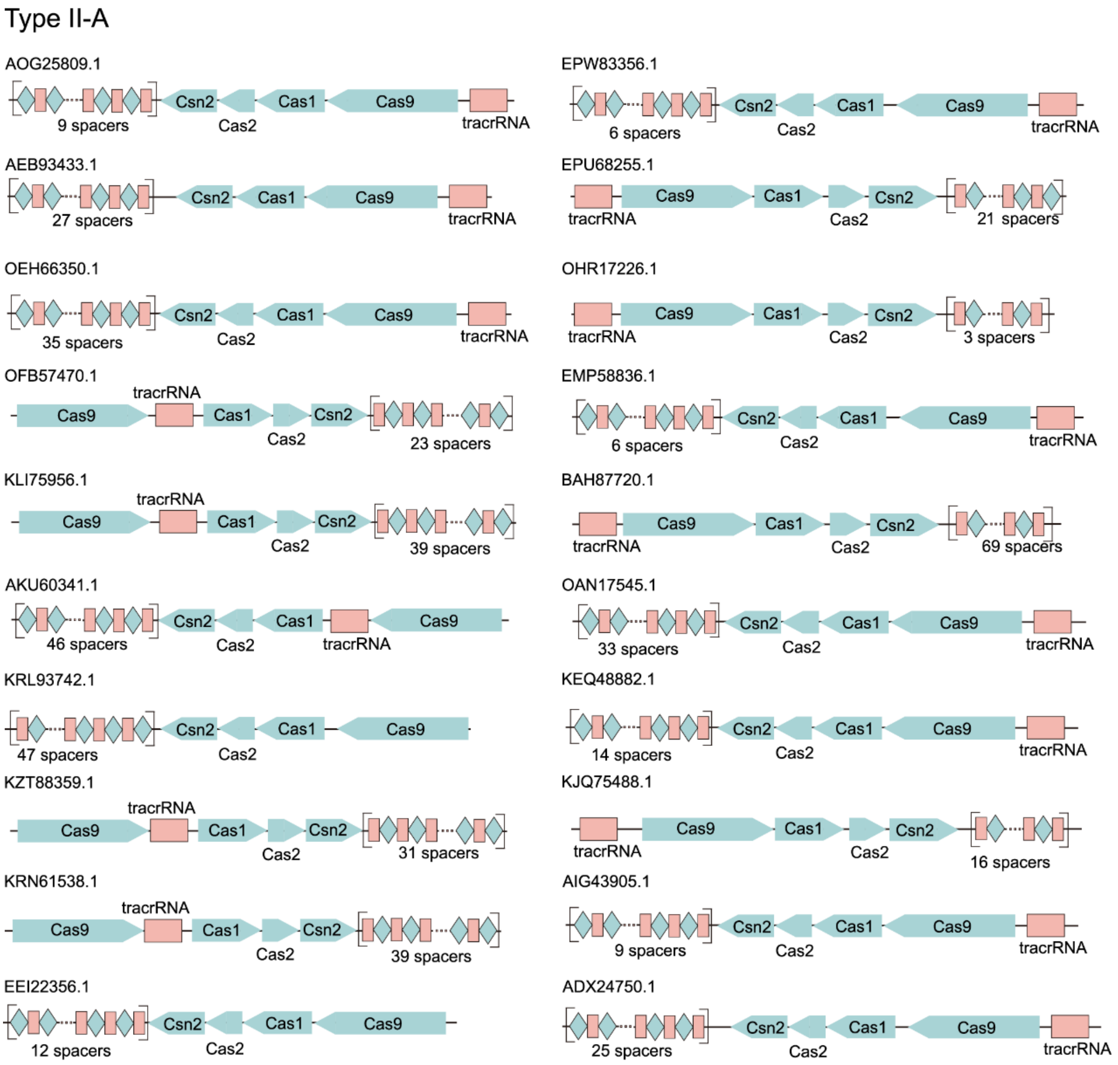

Supplementary Fig. 2. The 42 CRISPR-Cas9 loci examined in this study (part 1). Each CRISPR array was separated by square brackets. In each CRISPR array, the box in salmon indicates the crRNA while the diamond in cyan indicates the protospacer (spacer). The spacer number in the CRISPR array was shown below the CRISPR array.

Type II-A (continued)

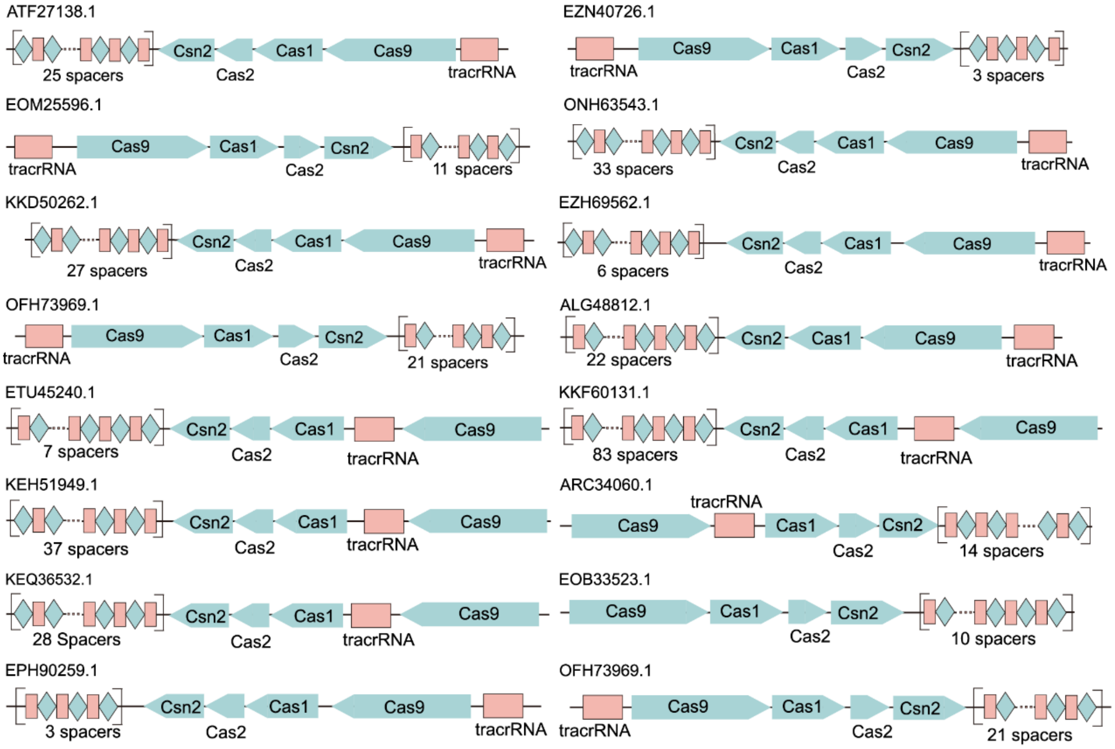

Type II-C

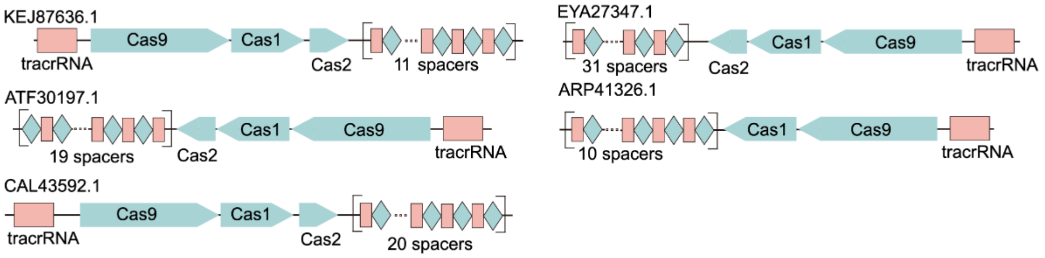

Supplementary Fig. 3. The 42 CRISPR-Cas9 loci examined in this study (part 2). Each CRISPR array was separated by square brackets. In each CRISPR array, the box in salmon indicates the crRNA while the diamond in cyan indicates the protospacer (spacer). The spacer number in the CRISPR array was shown below the CRISPR array.

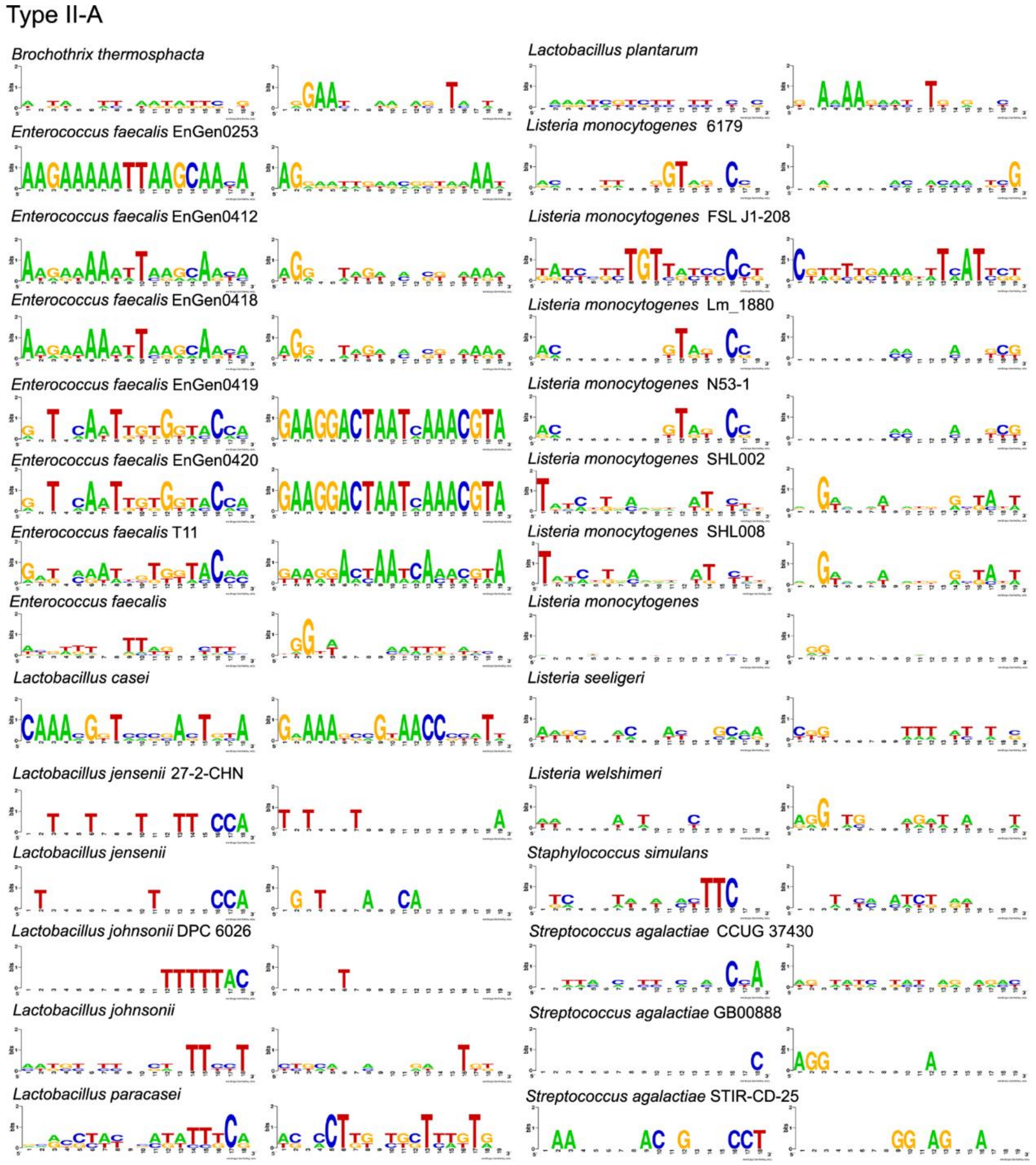

Supplementary Fig. 4. Predicted PAMs of the 42 CRISPR-Cas9 systems (part 1). The different directions of protospacers were tested. For each small panel, left, 5’ to 3’; right, 3’ to 5’.

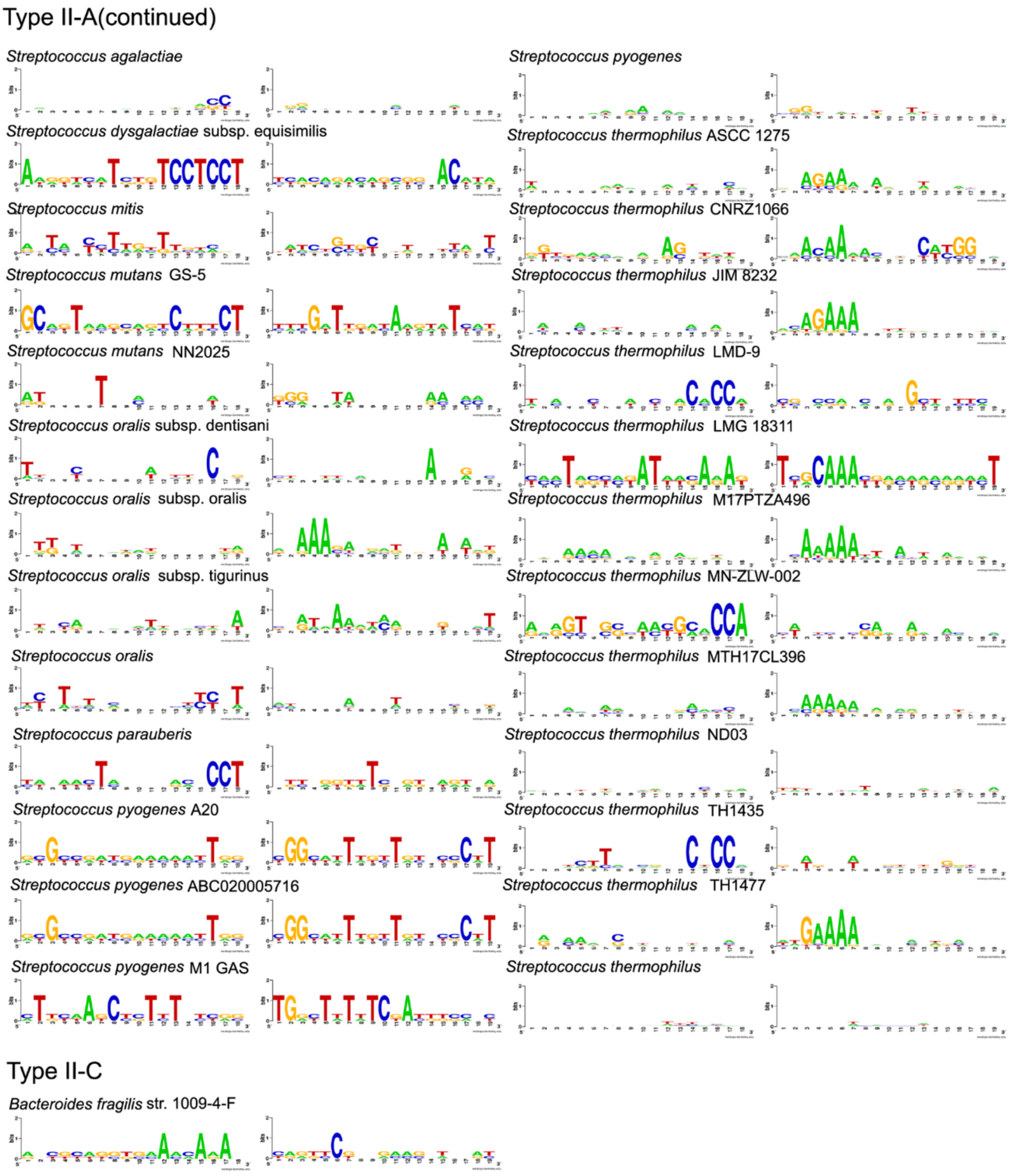

Supplementary Fig. 5. Predicted PAMs of the 42 CRISPR-Cas9 systems (part 2). The different directions of protospacers were tested. For each small panel, left, 5' to 3'; right, 3' to 5'.

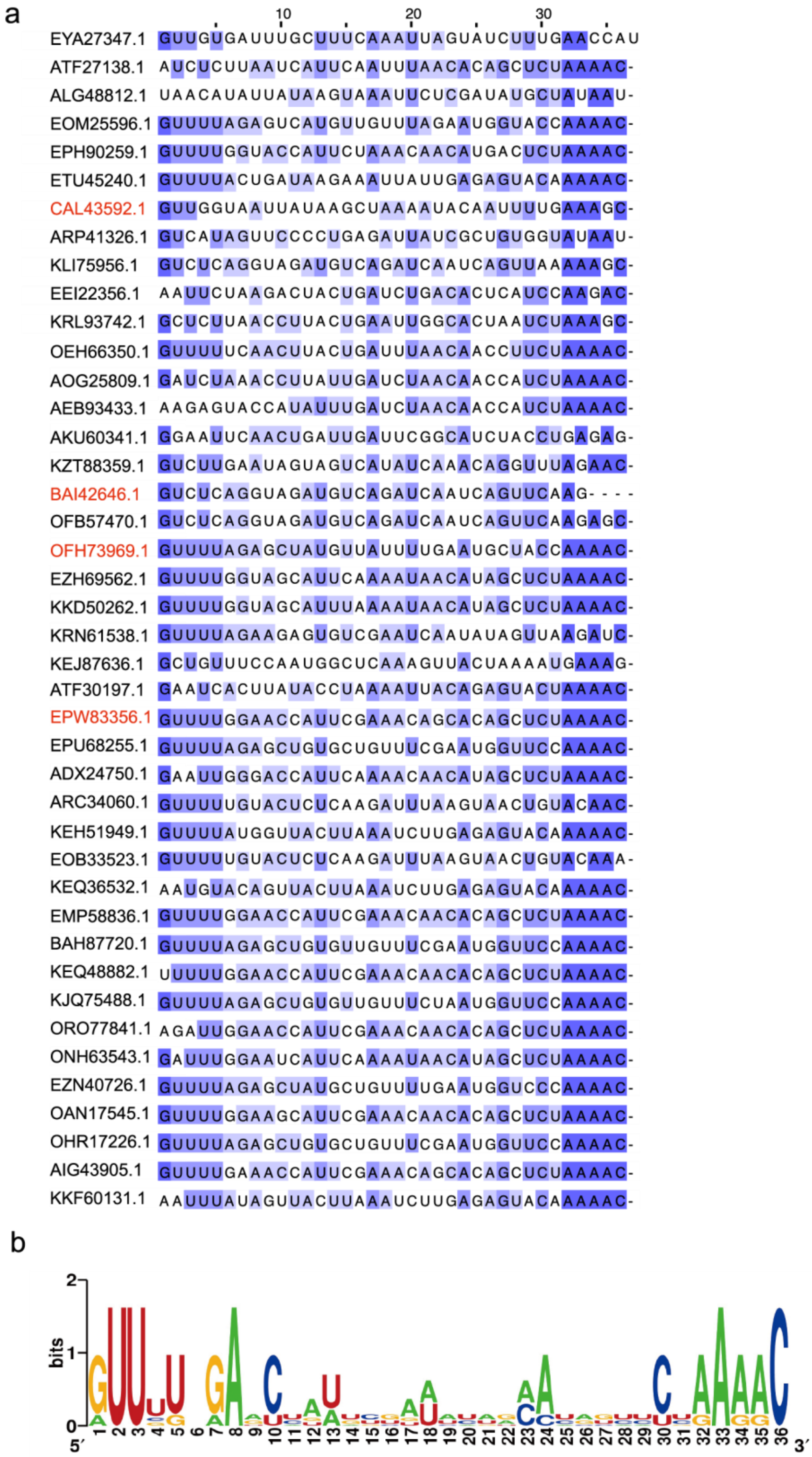

Supplementary Fig. 6. Analysis of the crRNA sequences of the 42 CRISPR-Cas9 systems. a, The alignment of 42 candidates' crRNAs. The deeper color indicates more conserved sequences. The four candidates further tested were labeled in salmon. **b,** The WebLogo of aligned 42 candidates' crRNAs.

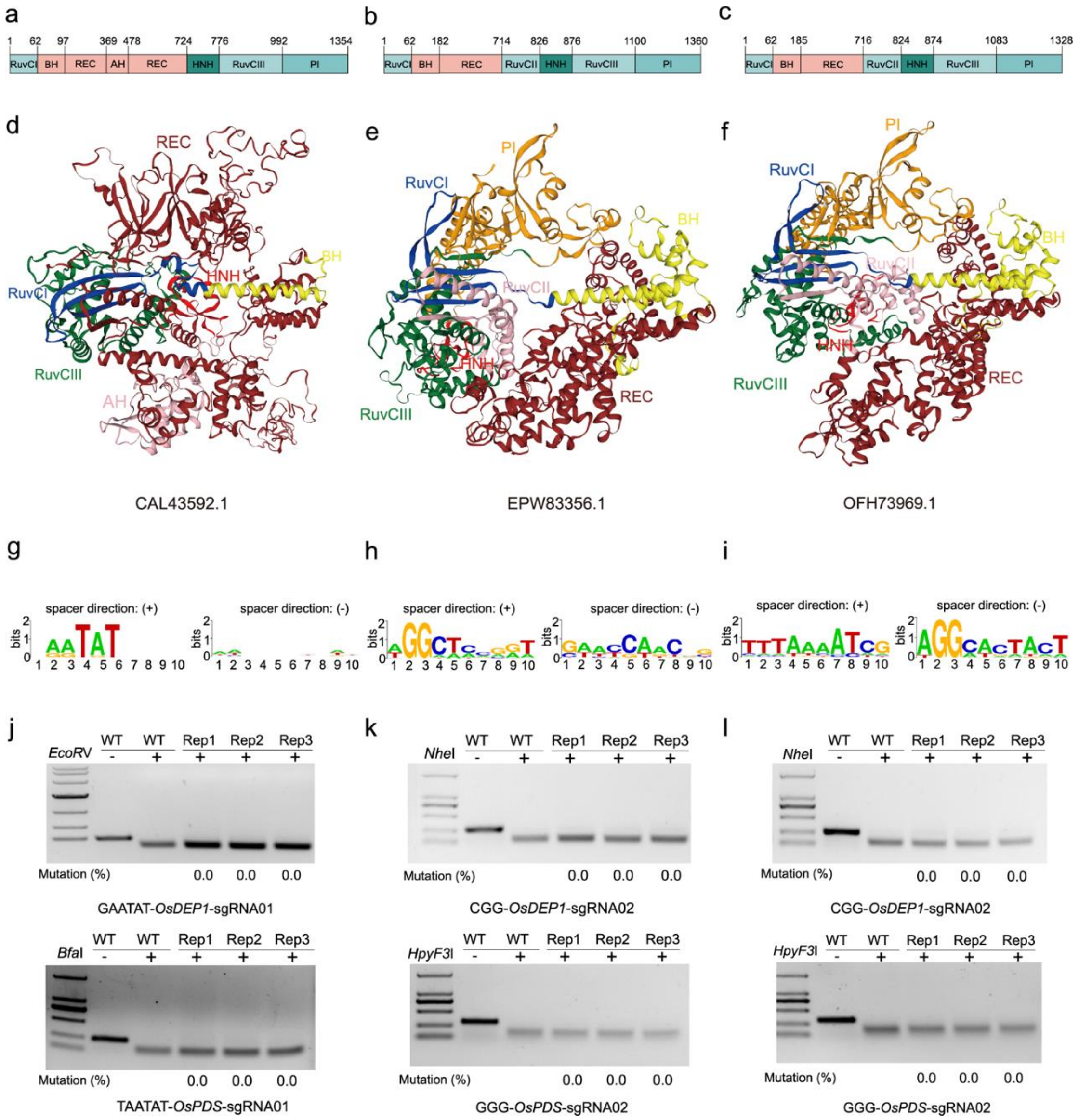

Supplementary Fig. 7. Structures, PAMs, and preliminary testing of three CRISPR-Cas9 systems in rice cells. a-c, The domain annotation of three new Cas9 nucleases. **d-f,** The protein structures of three new Cas9 nucleases. **g-i,** The predicted PAMs of three new Cas9 nucleases. **j-l,** The RFLP results of three new Cas9 nucleases at rice endogenous loci with predicted PAMs. The RFLP assays were repeated 3 times independently with the same results. Source data are provided as a Source Data file.

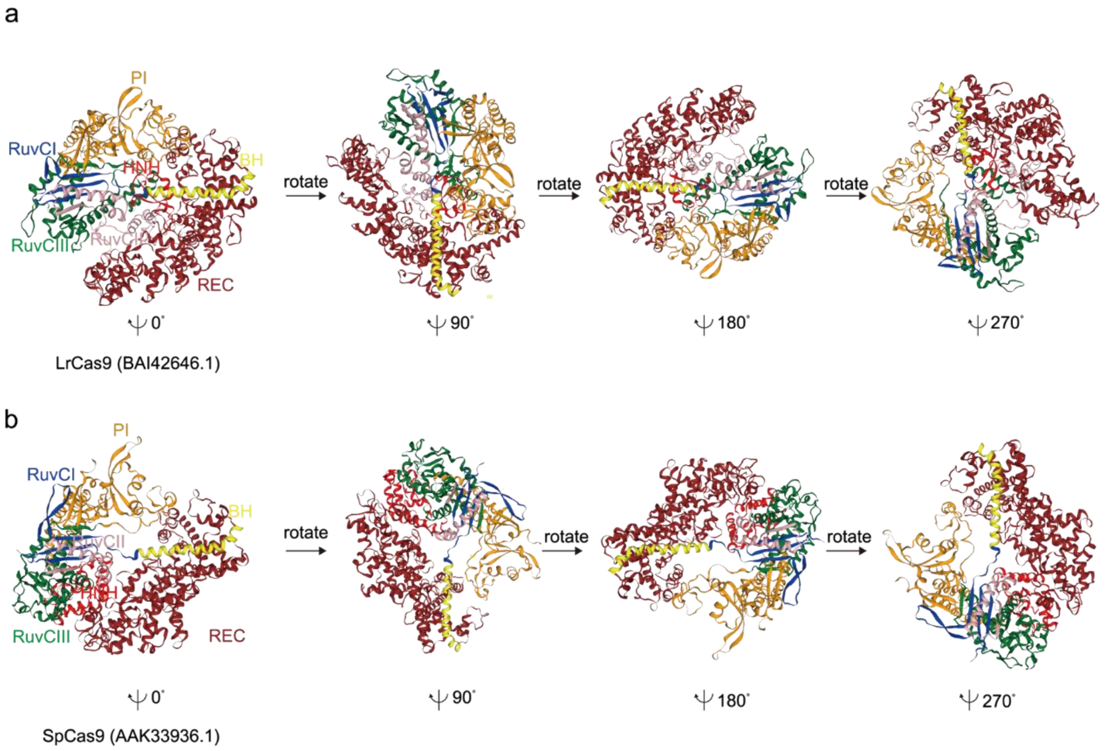

Supplementary Fig. 8. The structural front views of rotated LrCas9 and SpCas9 proteins. a, The protein structure of LrCas9 that rotated 90°, 180°, 270°. **b,** The protein structure of SpCas9 that rotated 90°, 180°, 270°. The protein structures were predicted by SWISS-MODEL.

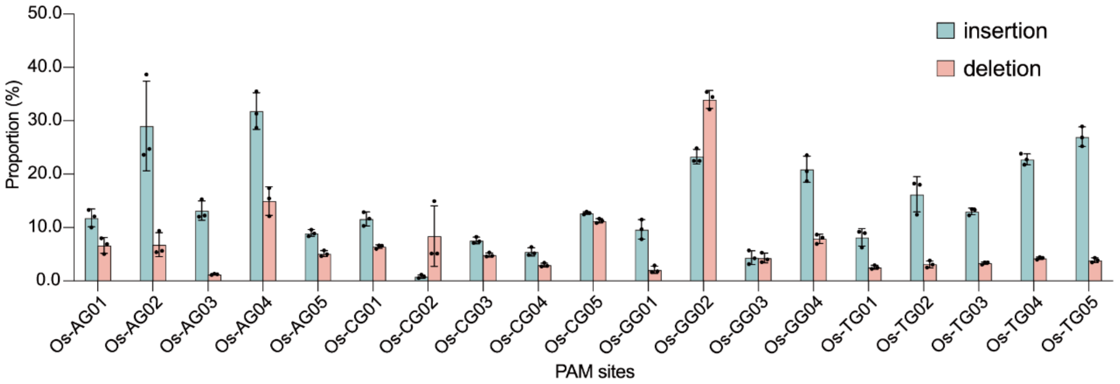

Supplementary Fig. 9. The insertion and deletion proportions by LrCas9 at 19 rice loci. Each dot represents a biological replicate. Each assay contains three independent experiments (*n*=3). Data are presented as mean values +/- SD. Source data are provided as a Source Data file.

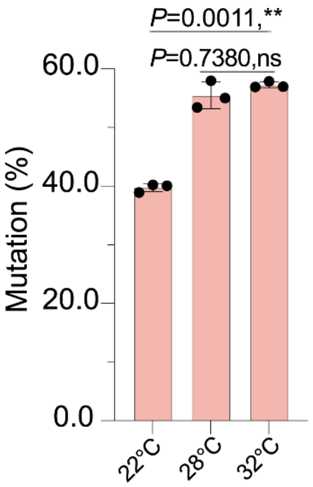

Supplementary Fig. 10. Genome editing by LrCas9 in rice protoplasts at Os-GG02 site at three different temperatures. The data were analyzed by one-way ANOVA with Dunnett's multiple comparisons tests. Each dot represents a biological replicate. Each target contains three biological replicates (*n*=3). Data are presented as mean values +/- SD. ns, $P>0.05$; **, $P<0.01$. Source data are provided as a Source Data file.

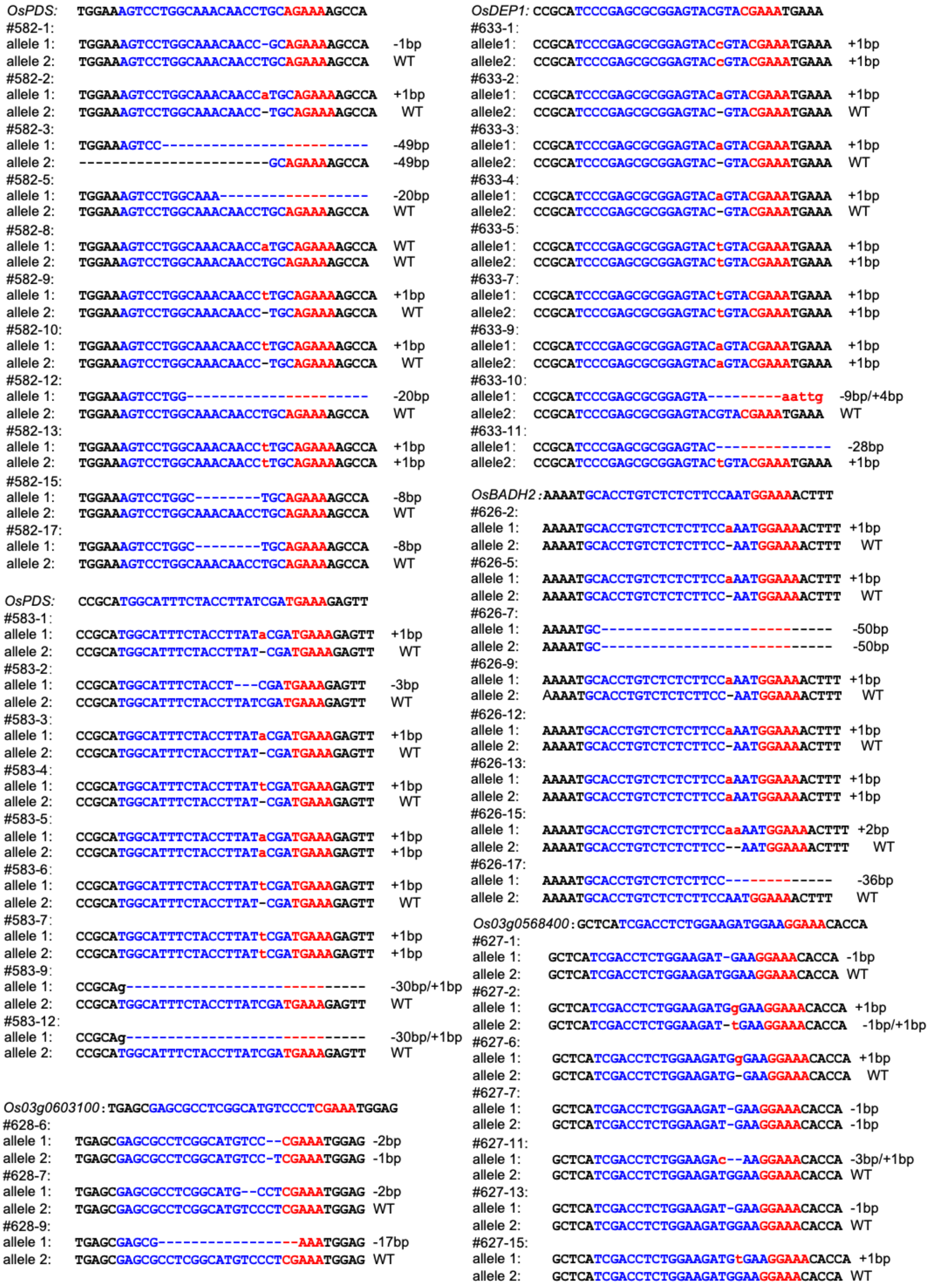

Supplementary Fig. 11. Genotype of LrCas9 mediated singular genome editing in rice T0 lines. The genotypes of rice T0 lines by LrCas9 editing with one sgRNA at a time. The protospacer sequence was labeled in blue, and the PAM sequence was labeled in red. The lowercase in red indicated nucleotide insertion, and the hyphen indicated nucleotide deletion.

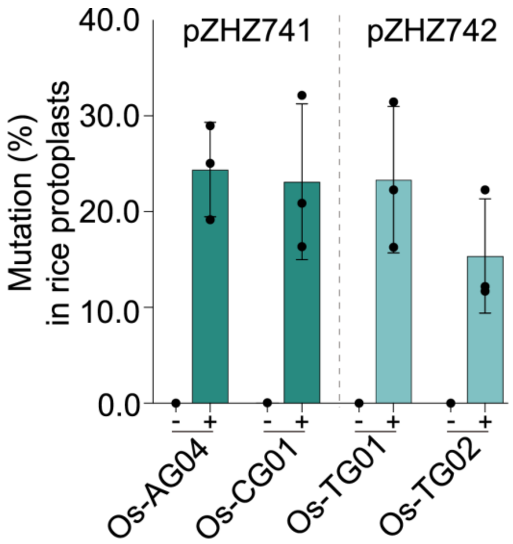

Supplementary Fig. 12. Testing LrCas9 for multiplexed genome editing in rice protoplasts. Each dot represents a biological replicate. Each target contains three biological replicates (*n*=3). Data are presented as mean values +/- SD. Source data are provided as a Source Data file.

```
OsPDS TGGAAAGTCCTGGCAAACAACCTGCAGAAAAGCCA
#741-6:
allele 1: TGGAAAGTCCTGG-------------GAAAAGCCA   -12bp
allele 2: TGGAAAGTCCTGGCAAACAACCTGCAGAAAAGCCA   WT
#741-18:
allele 1: TGGAAAGTCCTGGCAAACAACCTGCaAGAAAAGCCA   +1bp
allele 2: TGGAAAGTCCTGGCAAACAACCTGCAGAAAAGCCA   WT
#741-19:
allele 1: TGGAAAGTCCTGGCAAACAACCtTGCAGAAAAGCCA   +1bp
allele 2: TGGAAAGTCCTGGCAAACAACC-TGCAGAAAAGCCA   WT
#741-20:
allele 1: TGGAAAGTCCTGGCAAACAACCtTGCAGAAAAGCCA   +1bp
allele 2: TGGAAAGTCCTGGCAAACAACC-TGCAGAAAAGCCA   WT
#741-21:
allele 1: TGGAAAGTCCTGGCAAACAACCtTGCAGAAAAGCCA   +1bp
allele 2: TGGAAAGTCCTGGCAAACAAC--TGCAGAAAAGCCA   -1bp
#741-22:
allele 1: TGGAAAGTCCTGGCAAACAACCaTGCAGAAAAGCCA   +1bp
allele 2: TGGAAAGTCCTGGCAAACAACCaTGCAGAAAAGCCA   +1bp
#741-23:
allele 1: TGGAAAGTCCTGGCAAACAACCtTGCAGAAAAGCCA   +1bp
allele 2: TGGAAAGTCCTGGCAAACAAC--TGCAGAAAAGCCA   -1bp
```

```
OsDEP1 CCGCATCCCGAGCGCGGAGTACGTACGAAATGAAA
#741-6:
allele 1: CCGCATCCCGAGCGCGGAGTACtGTACGAAATGAAA   +1bp
allele 2: CCGCATCCCGAGCGCGGAGTACtGTACGAAATGAAA   +1bp
#741-18:
allele 1: CCGCATCCCGAGCGCGGAGTAC-GTAC---ATGAAA   -3bp
allele 2: CCGCATCCCGAGCGCGGAGTACaGTACGAAATGAAA   WT
#741-19:
allele 1: CCGCATCCCGAGCGCGGAGTAC---ACGAAATGAAA   -2bp
allele 2: CCGCATCCCGAGCGCGGAGTACaGTACGAAATGAAA   +1bp
#741-20:
allele 1: CCGCATCCCGAGCGCGGAGTAC---ACGAAATGAAA   -2bp
allele 2: CCGCATCCCGAGCGCGGAGTACaGTACGAAATGAAA   +1bp
#741-21:
allele 1: CCGCATCCCGAGCGCGGAGTAC--ACGAAATGAAA   -2bp
allele 2: CCGCATCCCGAGCGCGGAGTACaGTACGAAATGAAA   +1bp
#741-22:
allele 1: CCGCATCCCGAGCGCGGAGTAC--ACGAAATGAAA   -2bp
allele 2: CCGCATCCCGAGCGCGGAGTACaGTACGAAATGAAA   +1bp
#741-23:
allele 1: CCGCATCCCGAGCGCGGAGTAC--ACGAAATGAAA   -2bp
allele 2: CCGCATCCCGAGCGCGGAGTACaGTACGAAATGAAA   +1bp
```

```
OsPDS      AAGAGCTAGCCAAGCTATTTCCTGATGAAATTGCT
#742-2:
allele 1:  AAGAGCTAGCCAAGCTATTTCCTGAtTGAAATTGCT   +1bp
allele 2:  AAGAGCTAGCCAAGCTATTTCCTG-cTGAAATTGCT   -1bp/+1bp
#742-3:
allele 1:  AAGAGCTAGCCAAGCTATTTCCTGAaTGAAATTGCT   +1bp
allele 2:  ----------------------TGA-TGAAATTGCT   -2496bp
#742-9:
allele 1:  AAGAGCTAGCCAAGCTATTTCCtTGATGAAATTGCT   +1bp
allele 2:  AAGAGCTAGCCAAGCTATTTCCtTGATGAAATTGCT   +1bp
#742-10:
allele 1:  AAGAGCTAGCCAAGCTATTTCCtTGATGAAATTGCT   +1bp
allele 2:  AAGAGCTAGCCAAGCTATTTCCtTGATGAAATTGCT   +1bp
#742-11:
allele 1:  AAGAGCTAGCCAAGCTATTTCCtTGATGAAATTGCT   +1bp
allele 2:  AAGAGCTAGCCAAGCTATTTCCaTGATGAAATTGCT   +1bp
#742-12:
allele 1:  AAGAGCTAGCCAAGCTATTTCCtTGATGAAATTGCT   +1bp
allele 2:  -----------------------TGATGAAATTGCT   -2496bp
#742-14:
allele 1:  AAGAGCTAGCCAAGCTATTTCCaaTGATGAAATTGCT   +2bp
allele 2:  AAGAGCTAGCCAAGCTATTTCCt-TGATGAAATTGCT   +1bp
#742-16:
allele 1:  AAGAGCTAGCCAAGCTATTTCCaTGATGAAATTGCT   +1bp
allele 2:  AAGAGCTAGCCAAGCTATTTCCtTGATGAAATTGCT   +1bp
#742-17:
allele 1:  AAGAGCTAGCCAAGCTATTTCCtTGATGAAATTGCT   +1bp
allele 2:  AAGAGCTAGCCAAGCTATTTCCtTGATGAAATTGCT   +1bp
#742-18:
allele 1:  AAGAGCTAGCCAAGCTATTTCCtTGATGAAATTGCT   +1bp
allele 2:  -----------------------TGATGAAATTGCT   -2496bp
#742-19:
allele 1:  AAGAGCTAGCCAAGCTATTTCCtTGATGAAATTGCT   +1bp
allele 2:  AAGAGCTAGCCAAGCTATTTCCtTGATGAAATTGCT   +1bp
#742-21:
allele 1:  AAGAGCTAGCCAAGCTATTTCCtTGATGAAATTGCT   +1bp
allele 2:  AAGAGCTAGCCAAGCTATTTCCaTGATGAAATTGCT   +1bp
#742-22:
allele 1:  AAGAGCTAGCCAAGCTATTTCCaTGATGAAATTGCT   +1bp
allele 2:  AAGAGCTAGCCAAGCTATTTCCaTGATGAAATTGCT   +1bp
#742-23:
allele 1:  AAGAGCTAGCCA------------------TTGCT   -18bp
allele 2:  AAGAGCTAGCCAAGCTATTT---GATGAAATTGCT   -3bp
#742-24:
allele 1:  AAGAGCTAGCCA------------------TTGCT   -18bp
allele 2:  AAGAGCTAGCCAAGCTATTT---GATGAAATTGCT   -3bp
#742-25:
allele 1:  AAGAGCTAGCCA------------------TTGCT   -18bp
allele 2:  AAGAGCTAGCCAAGCTATTT---GATGAAATTGCT   -3bp
#742-27:
allele 1:  AAGAGCTAGCCA------------------TTGCT   -18bp
allele 2:  AAGAGCTAGCCAAGCTATTT---GATGAAATTGCT   -3bp
```

```
OsPDS      CCGCATGGCATTTCTACCTTATCGATGAAAGAGTT
#742-2:
allele 1:  CCGCATGGCATTTCTACCTTATtCGATGAAAGAGTT   +1bp
allele 2:  CCGCATGGCATTTCTACCTTAT-CGATGAAAGAGTT   wt
#742-3:
allele 1:  CCGCATGGCATTTCTACCTTgc------AAGAGTT   +2bp/-8bp
allele 2:  ---------------------CGATGAAAGAGTT   -2496bp
#742-9:
allele 1:  CCGCATGGCATTTCTACCTTATtCGATGAAAGAGTT   +1bp
allele 2:  CCGCATGGCATTTCTACCTTATtCGATGAAAGAGTT   +1bp
#742-10:
allele 1:  CCGCATGGCATTTCTACCTTATtCGATGAAAGAGTT   +1bp
allele 2:  CCGCATGGCATTTCTACCTTAT-CGATGAAAGAGTT   wt
#742-11:
allele 1:  CCGCATGGCATTTCTACCTTATtCGATGAAAGAGTT +1bp
allele 2:  CCGCATGGCATTTCTACCTTATaCGATGAAAGAGTT +1bp
#742-12:
allele 1:  ---------------------CGATGAAAGAGTT -2496bp
allele 2:  CCGCATGGCATTTCTACCTTATCGATGAAAGAGTT   wt
#742-14:
allele 1:  CCGCATGGCATTTCTAtCTTATCGATGAAAGAGTT   -1bp/+1bp
allele 2:  CCGCATGGCATTTCTACCTTATCGATGAAAGAGTT   wt
#742-16:
allele 1:  ---------------------CGATGAAAGAGTT -2496bp
allele 2:  CCGCATGGCATTTCTACCTTATCGATGAAAGAGTT   wt
#742-17:
allele 1:  CCGCATGGCATTTCTAtCTTATCGATGAAAGAGTT   -1bp/+1bp
allele 2:  ---------------------CGATGAAAGAGTT   -2700bp
#742-18:
allele 1:  CCGCATGGCATTTCTAtCTTATCGATGAAAGAGTT   -1bp/+1bp
allele 2:  CCGCATGGCATTTCTACCTTATCGATGAAAGAGTT   wt
#742-19:
allele 1:  CCGCATGGCATTTCTACCTTATtCGATGAAAGAGTT   +1bp
allele 2:  CCGCATGGCATTTCTACCTTAT-CGATGAAAGAGTT   wt
#742-21:
allele 1:  CCGCATGGCATTTCTAtCTTATCGATGAAAGAGTT   -1bp/+1bp
allele 2:  CCGCATGGCATTTCTACCTTATCGATGAAAGAGTT   wt
#742-22:
allele 1:  CCGCATGGCATTTCTAtCTTATCGATGAAAGAGTT   -1bp/+1bp
allele 2:  CCGCATGGCATTTCTACCTTATCGATGAAAGAGTT   wt
#742-23:
allele 1:  CCGCATGGCATTTCTAtCTTATCGATGAAAGAGTT   -1bp/+1bp
allele 2:  CCGCATGGCATTTCTACCTTATCGATGAAAGAGTT   wt
#742-24:
allele 1:  AAGAGCTAGCCA------------------TTGCT   -18bp
allele 2:  AAGAGCTAGCCAAGCTATTT---GATGAAATTGCT   -3bp
#742-25:
allele 1:  AAGAGTGGCATTTCTACCTTATtCGATGAAATTGCT   +1bp
allele 2:  AAGAGCTAGCCAAGCTATTTATtCGATGAAATTGCT   +1bp
#742-27:
allele 1:  AAGAGTGGCATTTCTACCTTATtCGATGAAATTGCT   +1bp
allele 2:  AAGAGCTAGCCAAGCTATTTATtCGATGAAATTGCT   +1bp
```

Supplementary Fig. 13. Genotypes of LrCas9 mediated multiplexed genome editing in rice T_0 lines. a, The genotypes in rice T_0 lines by LrCas9 with multiplexed genome editing construct pZHZ741. **b,** The genotypes in rice T_0 lines by LrCas9 with multiplexed genome editing construct pZHZ742. The protospacer sequence was labeled in blue, and the PAM sequence was labeled in red. The lowercase in red indicated nucleotide insertion, and the hyphen indicated nucleotide deletion.

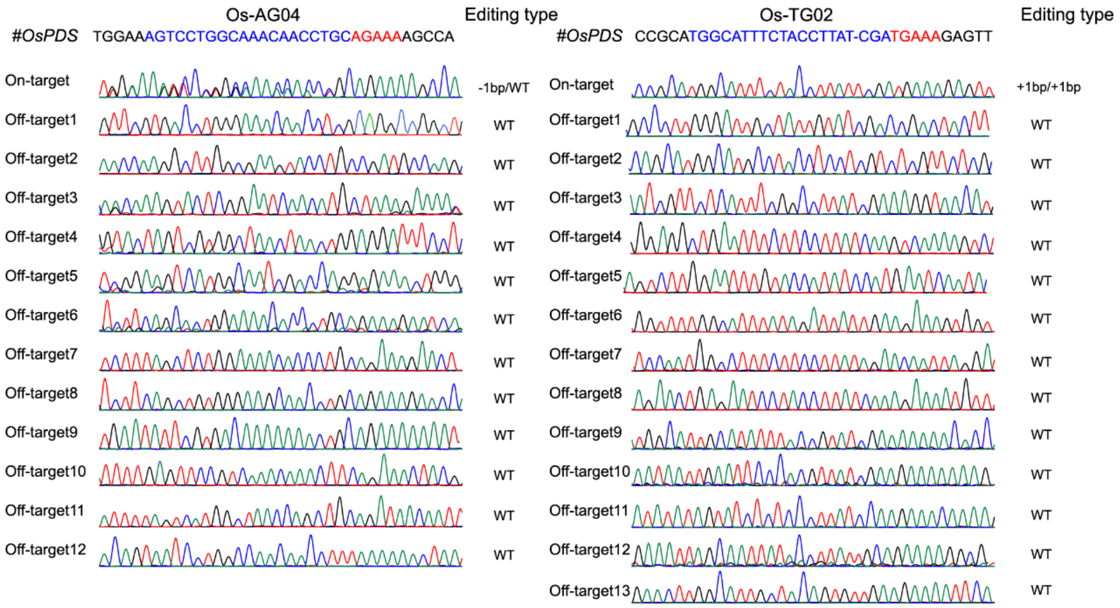

Supplementary Fig. 14. The Sanger sequencing results of edited T0 lines for all off-target sites that predicted by GUIDE-seq and CRISPR-GE.

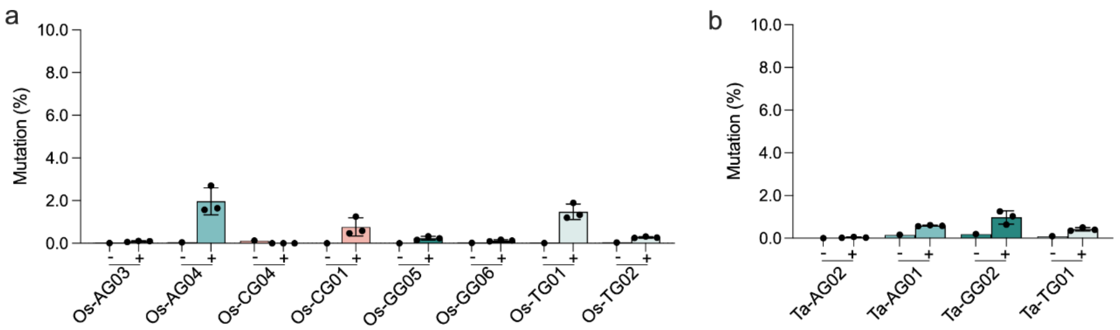

Supplementary Fig. 15. Indel byproduct frequency by LrCas9-CBE in rice and wheat protoplasts. a, The indel rates of LrCas9-CBE in rice protoplasts. **b,** The indel rates of LrCas9-CBE in wheat protoplasts. Each dot represents a biological replicate. Each target contains three biological replicates (*n*=3). Data are presented as mean values +/- SD. Source data are provided as a Source Data file.

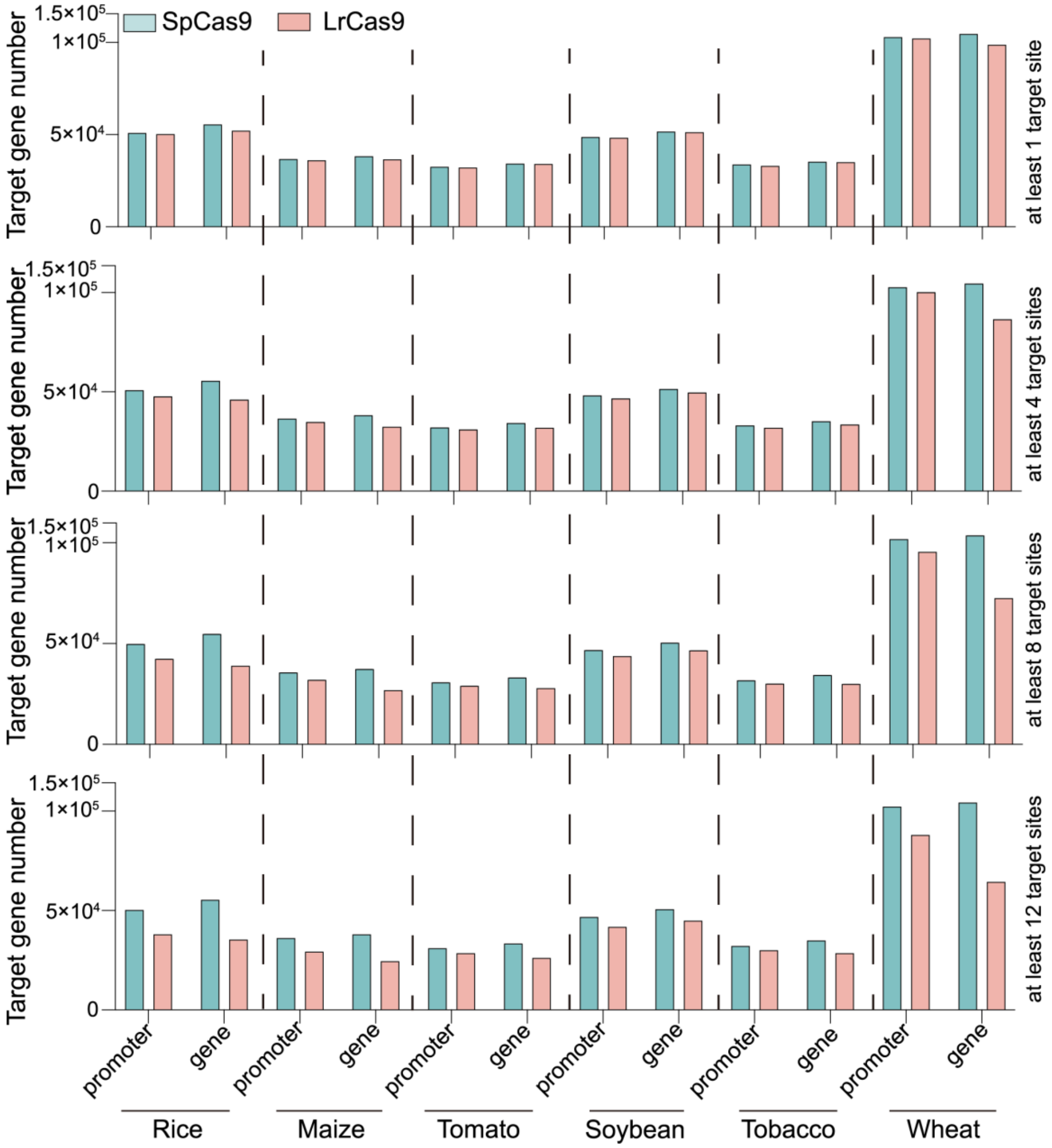

Supplementary Fig. 16. Targeting scope of SpCas9 and LrCas9 in six major crops. The analysis was done for four arbitrary conditions with different numbers of target sites defined per gene for each plant species. Source data are provided as a Source Data file.

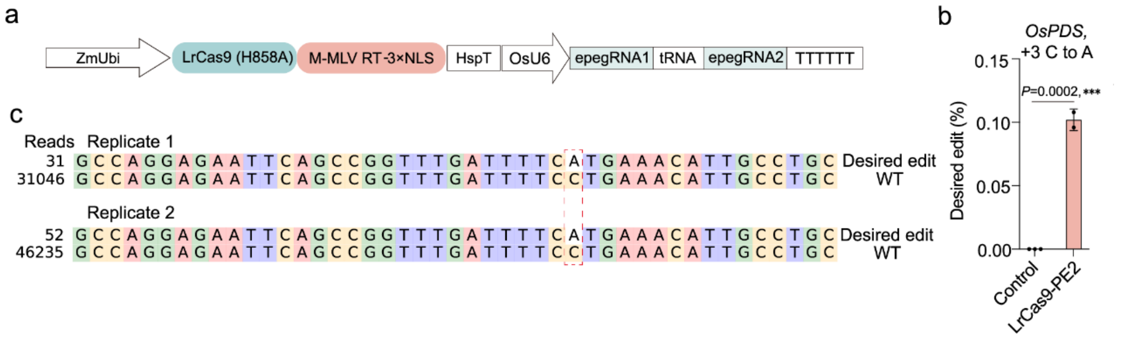

Supplementary Fig. 17. Prime editing by LrCas9-PE2 in rice protoplasts. a, The illustration of LrCas9 prime editor 2 (PE2). **b,** The desired edit ratio at *OsPDS* gene in rice protoplasts by deep-sequencing. The data were analyzed using two-tailed unpaired *t*-Test. Each dot represents a biological replicate. Each target contains two or three biological replicates (*n*=2 or *n*=3). Data are presented as mean values +/- SD. ***, *P*<0.001. **c,** The desired edit reads of LrCas9-PE2 in rice protoplasts with two replicates. The desired editing nucleotide are dash squared. Source data are provided as a Source Data file.

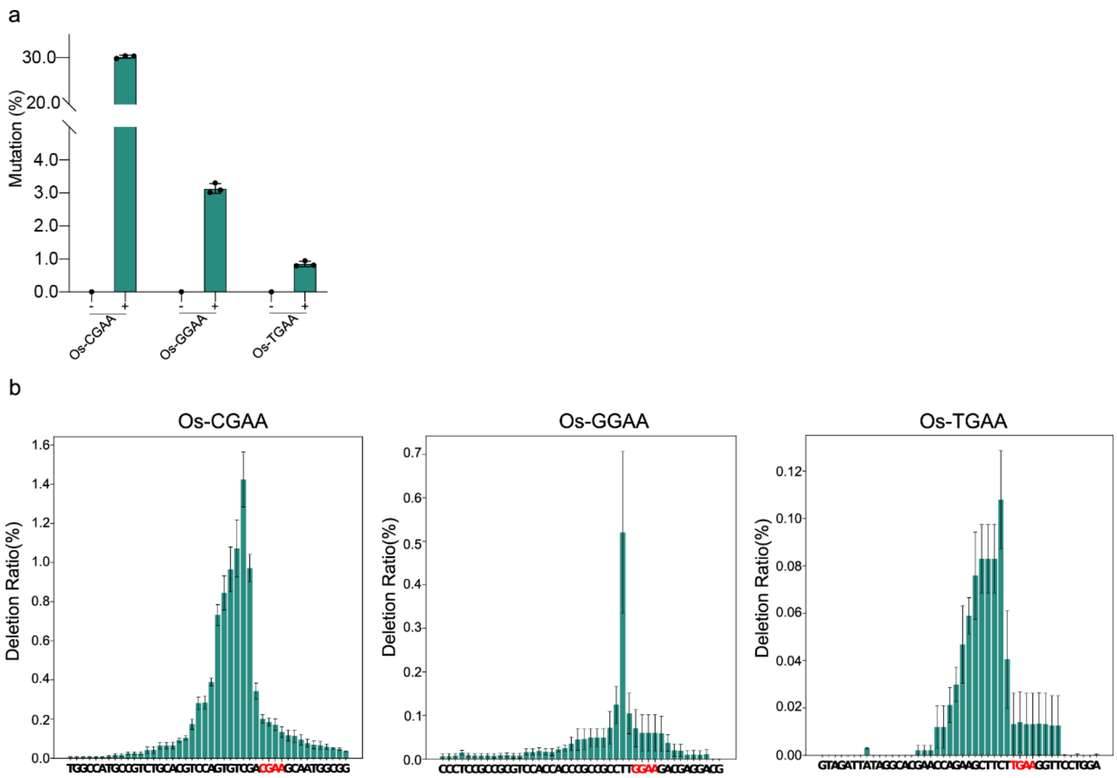

Supplementary Fig. 18. Genome editing efficiency at NGAA PAM sites by LrCas9 in rice protoplasts. a, The mutation rates of LrCas9 at 5’-NGAA-3’ sites in rice protoplasts. Each dot represents a biological replicate. Each target contains three biological replicates (*n*=3). Data are presented as mean values +/- SD. **b,** The deletion profile of LrCas9 at 5’-NGAA-3’ sites in rice protoplasts. The PAM was labeled with red. Source data are provided as a Source Data file.

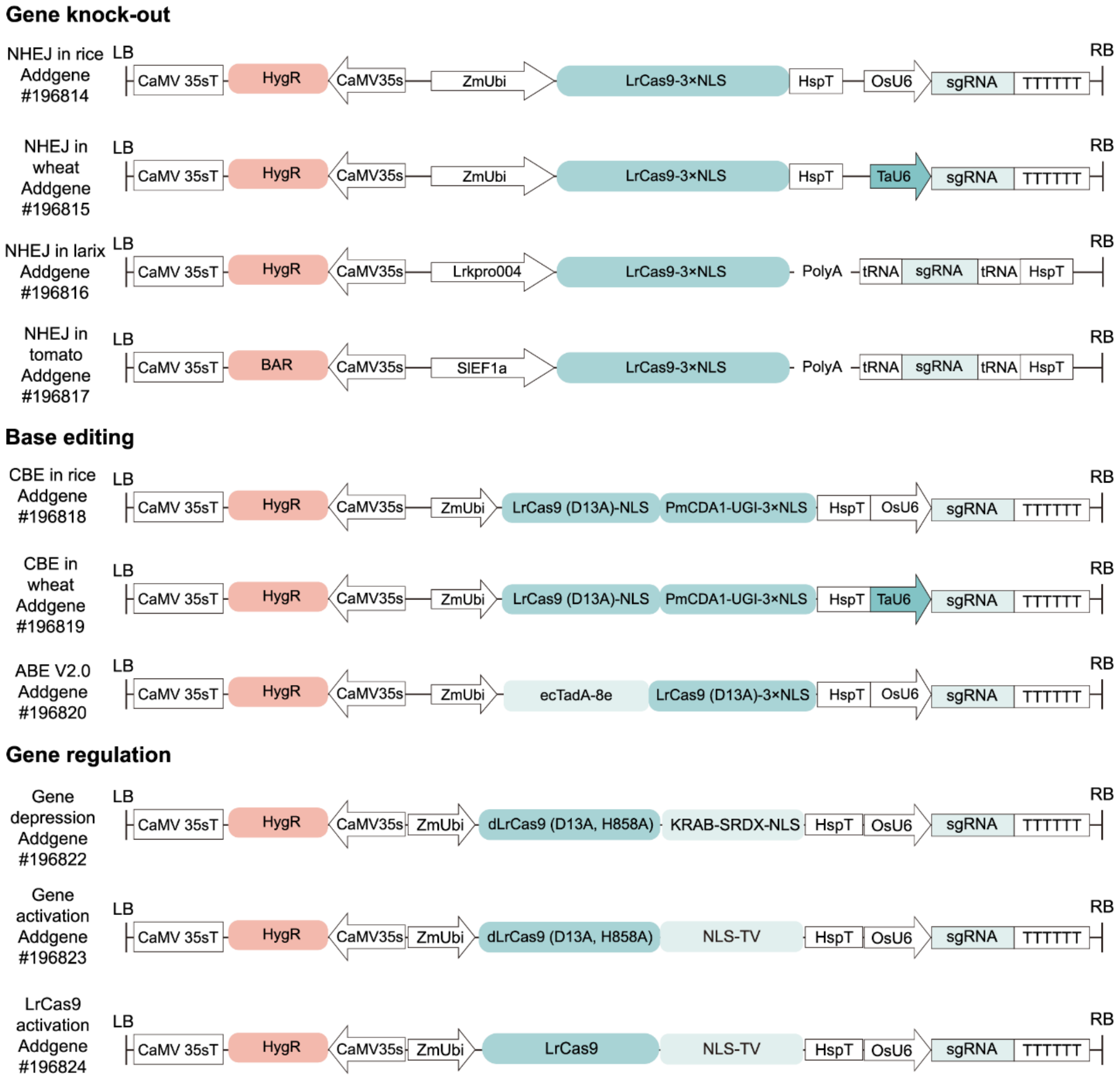

Supplementary Fig. 19. Plasmid maps for the CRISPR-LrCas9 genome engineering systems developed in this study. Note all these plasmids have been deposited to Addgene, with the Addgene numbers listed on the left side.
