# Supplementary material for: Efficient plant genome engineering using a probiotic sourced CRISPR-Cas9 system
Source: Nat Commun. 2023 Sep 29;14:6102. doi: 10.1038/s41467-023-41802-9 (PMC10541446; doi:10.1038/s41467-023-41802-9)
Supplement: Supplementary file 3 — Description of Additional Supplementary Files [file 41467_2023_41802_MOESM3_ESM.pdf]

Supplementary Data 1
The predicted CRISPR locus and PAM information in this study.

Supplementary Data 2
The protospacers and anti- protospacers detail of LrCas9 CRISPR array.

Supplementary Data 3
GUIDE-seq detected potential off-target sites.

Supplementary Data 4
Target sites in this study.

Supplementary Data 5
Oligos used in this study.
